# The influence of tantalum oxide nanoparticle on thermal conductivity and tear strength after addition on maxillofacial silicone

**DOI:** 10.12688/f1000research.159001.1

**Published:** 2025-02-11

**Authors:** saif Ali Mousa, Zainab Saleh Abdullah

**Affiliations:** 1 Department of Prosthodontic, College of Dentistry, University of Baghdad, Baghdad, Baghdad Governorate, 10011, Iraq

**Keywords:** Tantalum oxide (Ta2O5), VST-50F silicone elastomer, thermal conductivity, tear strength.

## Abstract

**Background:**

Maxillofacial prosthetic materials exhibit a range of qualities that vary considerably, including the hardness and stiffness of alloys and polymers, as well as the flexibility of elastomers and soft polymers. Silicone elastomers are the primary materials employed for maxillofacial prostheses due to their physical properties, which allow for adaptation to the movement of soft tissue. They also exhibit exceptional tensile and tear strength across a broad temperature range. The addition of nanoparticle enhance thermal and tear strength of maxillofacial silicon.

This study estimates the influence of addition tantalum oxide nanoparticle on thermal conductivity and tear strength after addition on maxillofacial silicone.

**Method:**

Tantalum oxide nanoparticles were added into VST-50F platinum silicone elastomer at two weight percentages: 1wt% and 1.5wt%. 60 specimens were prepared and classified into two groups: one control group for each property and two experimental groups. All collected data underwent statistical analysis by one-way ANOVA and Bonferroni’s multiple comparison test, with significance set at p < 0.05. The Shapiro-Wilk test and Bartlett’s test were employed to assess the normality and homogeneity of the data, respectively.

**Result:**

The one-way ANOVA test found a very significant difference between all groups, while Bonferroni’s test revealed a highly significant difference between the control and experimental groups. Thermal conductivity test found no significant difference between the 1wt% and 1.5wt% groups, however the tear strength test was highly significant.

**Conclusions:**

The addition tantalum oxide nano particles improve tear strength and thermal conductivity.

## Introduction

The branch of prosthodontics known as maxillofacial prosthetics is concerned with replacing and restoring both stomatognathic and related facial features with artificial replacements that may or may not be removed. ^1^

Using maxillofacial prostheses for facial restoration, these patients could improve their functional rehabilitation, restore their appearance, correct speech difficulties, and support their mental health, allowing them to resume their social connections. ^2, 3^

Over time, when the maxillofacial prosthesis touches human skin, it might absorb sweat and sebum. ^4^

The efficiency of maxillofacial prosthesis materials used for facial defect reconstruction is restricted by appropriate mechanical and physical properties, as well as the capacity to maintain these features over the prosthesis’s service life, as they must not be inflammatory or carcinogenic. Maxillofacial prostheses used to rehabilitate individuals with face deformities can deteriorate and discolor due to ambient exposure to ultraviolet (U.V.) radiation, air pollution, and increases in humidity. ^5^

The translucency of silicone is the primary advantage, facilitating color matching with the patient’s missing structure. Silicone exhibits biocompatibility, color stability, and controllability. Silicone exhibits exceptional thermal stability and maintains color fidelity when subjected to ultraviolet light, is aesthetically pleasing, biologically inert, and maintains physical and chemical qualities across a broad temperature range. ^6^

The primary disadvantage of silicon is its early deterioration due to a variety of variables, including handling, hygiene, air pollution, and temperature changes, even though VST-50 (R.T.V.) maxillofacial silicone has several promising qualities. Additionally, when different disinfection techniques are used as part of the prosthesis maintenance regimen, silicone’s extremely porous nature may cause discoloration. Additionally, when different disinfection techniques are used as part of the prosthesis maintenance regimen, silicone’s extremely porous nature may cause discoloration. ^7, 8^

Silicone prostheses can be cleaned using a variety of methods, including mechanical cleaning with manual brushing or hand washing with neutral soap and chemical cleaning with nontoxic disinfectants. ^9^

The main drawback of silicone material is the significant risk of early deterioration, which might manifest as lower tear strength, a reasonably fitting margin, material discoloration, and a modified texture. As a result, silicone elastomers are typically reinforced with fillers to improve their mechanical qualities. The efficiency of fillers is connected to their features, such as particle form and size, as well as the strength of filler-polymer interaction, which enhances the degree of crosslinking. ^10^

While materials with low heat conductivity are referred to as insulators, those with high thermal conductivity are considered good conductors. Elastomers’ mechanical qualities and thermal stabilities are enhanced by the fillers in addition to their thermal conductivity. ^11– 13^

The clinically significant tear strength property is paramount for achieving aesthetic results, precise marginal adaptation, and integrity, all of which are essential for maxillofacial prostheses to integrate seamlessly with surrounding tissues; thus, specialized margins are required to connect the prosthesis with the patient’s tissues. Nonetheless, slender edges yield exceedingly delicate margins that can fracture during processing. Insufficient tear strength may lead to margin degradation during the removal of facial prostheses for maintenance. Improved tear strength signifies that the prosthesis can be fabricated with a refined finishing line that blends naturally with the skin, concealing the prosthesis’s edge. ^14, 15^

The addition of nanoparticle-like tantalum oxide (Ta _2_O _5_) may improve the maxillofacial silicon’s tear strength, thermal conductivity, and other characteristics. Apatite has formed and nucleated on the surface of tantalum metal due to its bioactivity and affinity for phosphate groups. Furthermore, it has been discovered that tantalum oxide is bioactive, encouraging the formation of hydroxyapatite and the attachment of osteoblasts. Because of their great workability and fracture toughness, tantalum oxide and metal alloys containing tantalum have been used in the medical and dentistry fields. Because of these characteristics, tantalum is being researched for potential application in dentistry and has been added to materials made of polymers. ^16, 17^

**Hypothesis of study**: Thermal conductivity and tear strength do not vary by the addition of Ta _2_O _5_ nanoparticles, according to the null hypothesis (H0). Thermal conductivity and tear strength may be varied by the addition of Ta _2_O _5_ nanoparticles, according to the alternative hypothesis (H1).

## Methods

Tantalum oxide (Ta _2_O _5_) (Sky Spring Nanomaterials, U.S.A.) and VST-50F room temperature vulcanized silicone (Factor II Inc., U.S.A.) were used.

The Ta _2_O _5_ particles were confirmed to be at the nanoscale using a particle size analyzer, and the effective diameter was (70.2 nm).

### Pilot study

A pilot study was conducted on various percentages of nanoparticles incorporated into maxillofacial silicone to evaluate their effects, after which the optimal percentage enhancing tear strength and thermal conductivity will be determined. Following the identification of the two most suitable percentages of modified maxillofacial silicone from the pilot study, new specimens will be fabricated for utilization in the main investigation, employing the tests of tear strength and thermal conductivity.

### Specimen grouping

A total of 60 specimens were produced and classified into two groups according to the tests (tear strength and thermal conductivity), with each group comprising 30 specimens. Group A, designated as the control group, contained 0% Ta _2_O _5_ nanofiller, while groups B and C, referred to as experimental groups, contained 1 wt% and 1.5 wt% Ta _2_O _5_ nanofiller, respectively.

### Mold fabrication

Three transparent acrylic sheets (the matrix, bottom, and cover) with a thickness of 2 ± 0.05 mm were produced. The matrix sheet for the tear strength test was constructed with an unticked edge and a 90° angle on one side, featuring tab ends. In contrast, the thermal conductivity test matrix was designed with disco-shaped perforations measuring 40 mm in diameter and 2.5 mm in thickness. These were affixed to the bottom sheet using chloroform as an adhesive to prevent movement during the silicone pouring process. Utilizing CorelDraw 2020 software for mold design and employing a C.N.C. machine for fabrication. Clamps, screws, and nuts were additionally used for enhanced tightness at the peripheries. ^18^

### Fabrication and conditioning of the specimens

The specimens of the control subgroup were prepared and combined at a base-to-catalyst ratio of 10:1 (w/w), following the manufacturer’s guidelines. A vacuum mixer was employed to blend the mixture for 5 minutes at a speed of 140 ± 10 rpm at a vacuum of -10 bar (-28 inches Hg).

All specimens from the experimental subgroup were mixed in a manner analogous to the control subgroups, with the same mixing ratio but incorporating nano Ta _2_O _5_ filler with a clean spatula. The silicone elastomer base was incorporated and mixed without vacuum for three minutes to prevent powder suction, followed by activation of the vacuum mixer for seven minutes at a speed of 140 ± 10 rpm with a vacuum pressure of -10 bar to eliminate air bubbles. The mixture was subsequently permitted to cool for 5 minutes. The catalyst was subsequently incorporated into the base-filler combination and blended for five minutes. The mixture was put into the mold, and the cover was secured over it. The mold was secured using screws, nuts, and G-clamps. The mixture was allowed to cure at (23°C ± 2°C) for 4 hours per the manufacturer’s guidelines. The specimens were maintained at 20-25°C with 50 ± 10% humidity for 16 hours before testing, in accordance with ISO 23529:2016. ^18, 19^

### Experimental tests

**Test for tear strength**

The specimens, constructed in accordance with ISO 34-1 (2022) specifications, featured an unticked design with a 90° angle on one side and tab ends. They were subjected to testing using a universal testing machine to evaluate tear initiation at a stress concentration point positioned at the 90° apex. The thickness is 2 ± 0.2 mm. ^20^

The tear strength, measured in N/mm, was determined using the subsequent equation. (ISO 34-1, 2022):

Ts(Tear strength)=f/d−−−−−−−−−−−−(2−2)

Where:

f: The maximum force required for complete rupturing of the sample at the apex (kN). d: The average thickness of the tested specimen in (millimeters). ^20^

**Test for thermal conductivity**

All samples were prepared with the Hot Disc apparatus. The specimen must exhibit a flat and smooth surface, with disc samples measuring 40 mm in radius and 6 mm in thickness (ISO 22007-2, 2022). The transient plane heat source (T.P.S.) thermal conductivity tester device (model: Skz1061C) was used to perform the test (ISO 22007-2, 2022). This apparatus employs a double helical plane probe inscribed with alloy sheets. The plane probe must be positioned between two specimens and secured with a fastening. The probe functions as both a heat source and a sensor. The power bridge technique is employed to measure the specimen by detecting voltage variations on the probe. The acquired data is subsequently transmitted to computer software for analysis and processing, ultimately yielding thermal conductivity. The thermal conductivity was assessed in **(W/m.k.)**. ^21^

Where:

**W** represents watt.

**m** is meter.

**k** is kelvin.

### Fourier transforms infrared spectroscopy (FTIR)

FTIR (IRAffinity-1 laser product, Shimadzu, Japan) was employed to ascertain whether there was a chemical interaction between the silicone substance and the Ta _2_O _5_ nanoparticles. Three samples, one from each group, were examined. (Control, 1 wt% and 1.5 wt%). The resolution was at 400-4000 cm ^−1^.

### Statistical analysis

The study’s results were assessed using SPSS (Statistical Package for the Social Sciences, version 24) software, employing one-way ANOVA (Analysis of Variance) to compare the mean values of the examined groups. The significance of the difference between the two tested groups was assessed using Bonferroni’s multiple comparisons test. The Shapiro-Wilk test was employed to assess the normality of data distribution, while Bartlett’s test was utilized to evaluate the homogeneity of variances.

P values of >0.05 were considered statistically non-significant (N.S.), P values of ≤0.05 were considered statistically significant (S), and P values of ≤0.01 were considered highly significant.

## Results

FTIR Results: The addition of Ta _2_O _5_ did not alter the spectral range of VST-50F silicone, indicating no chemical interaction, as shown in Figure 3. One test sample was evaluated in order to compare it with the control group; the FTIR was simply utilized to verify whether or not there was a chemical reaction.

**Figure 1.**
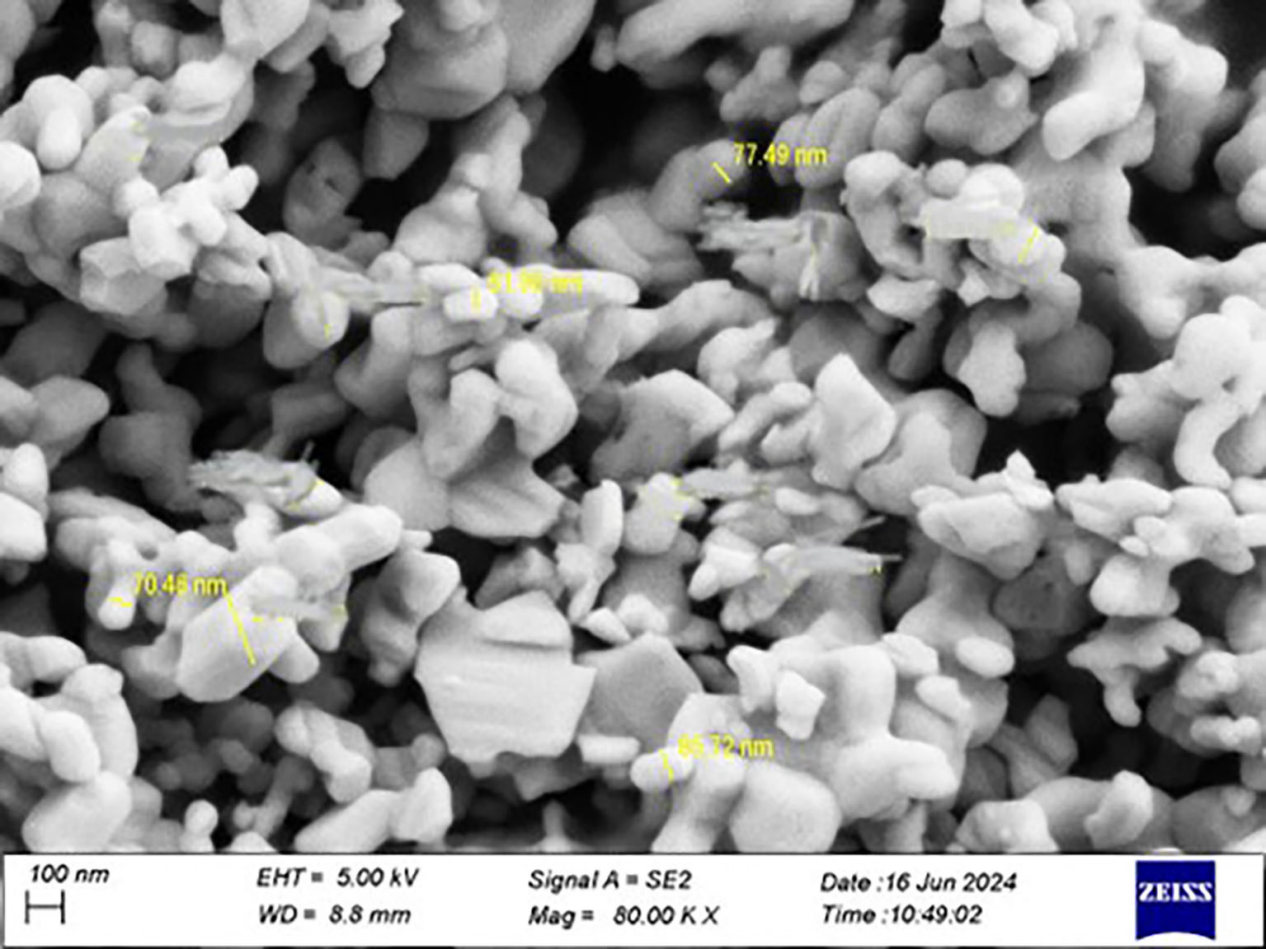
Particle size analyzer for tantalum oxide nanoparticle.

**Figure 2.**
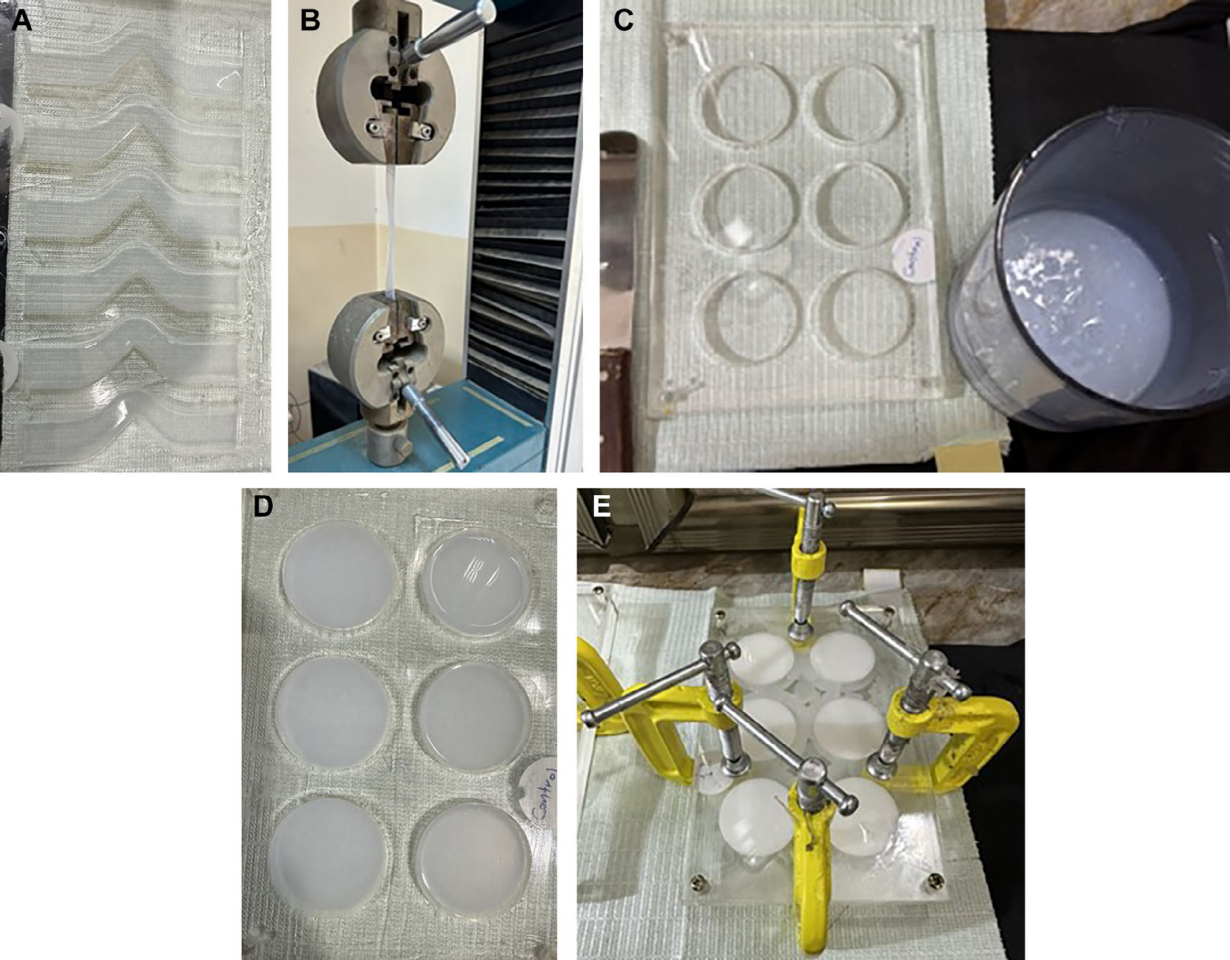
A: Samples poured in tear Mold. B: Sample tested in Universal testing machine. C: Material ready for poured in thermals mold. D: Samples for thermal test. E: The mold is secured with screws, nuts, and G-clamps after pouring the mixed silicone inside.

**Figure 3.**
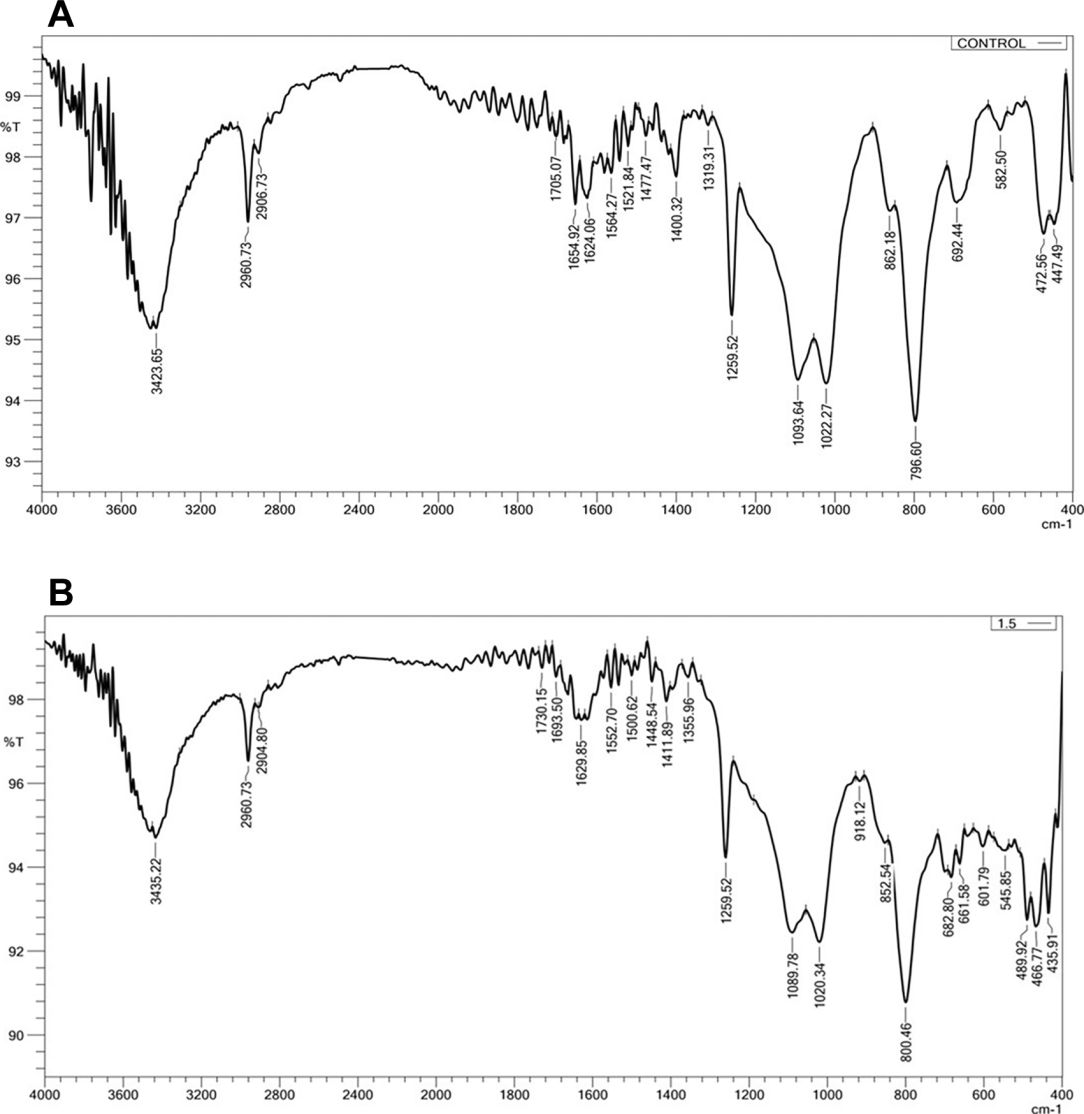
A, FTIR of control specimen; B, FTIR of 1.5 wt% Ta _2_O _5_ specimen, revealing there is no chemical interaction. The X-axis refers to Wavenumber cm-1, Y-axis refers to Transmittance %.

**Figure 4.**
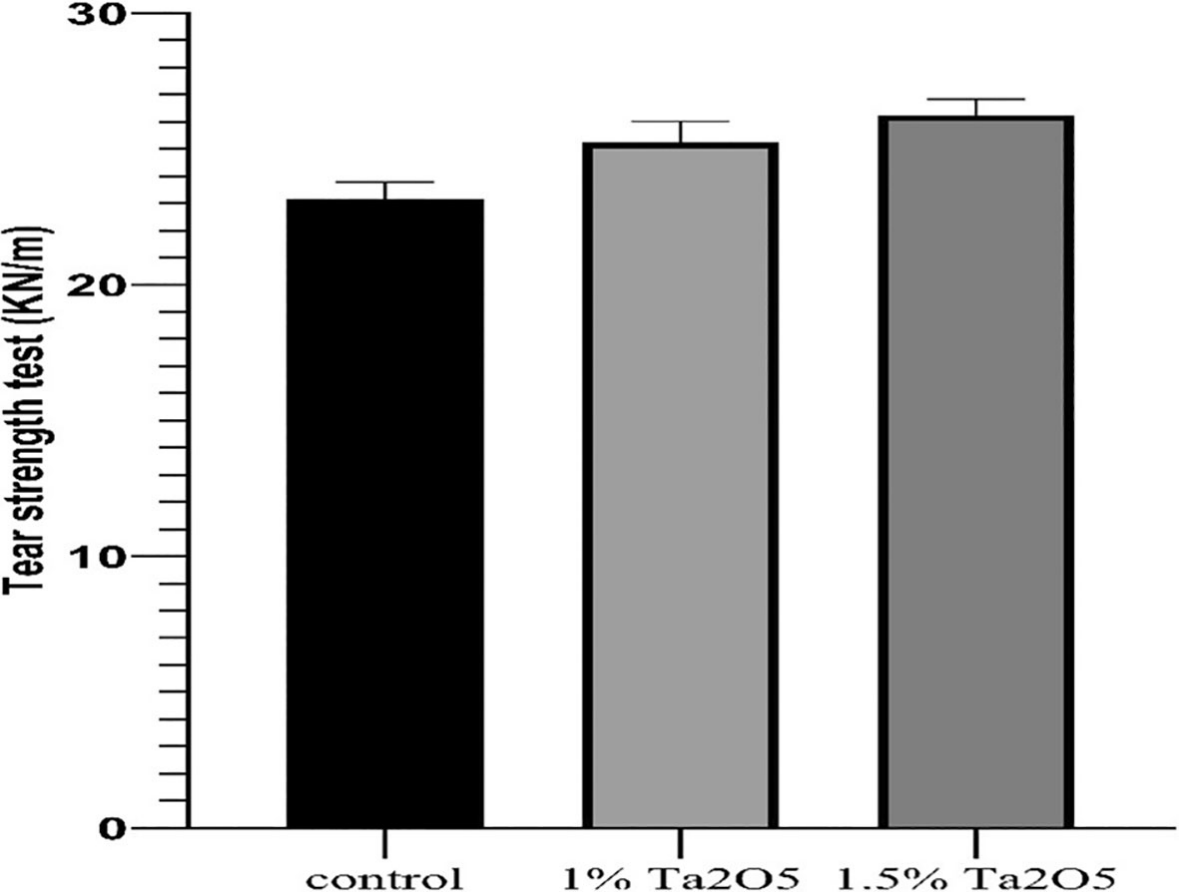
Bar chart of the mean and standard deviation of tear strength for studied groups.

**Figure 5.**
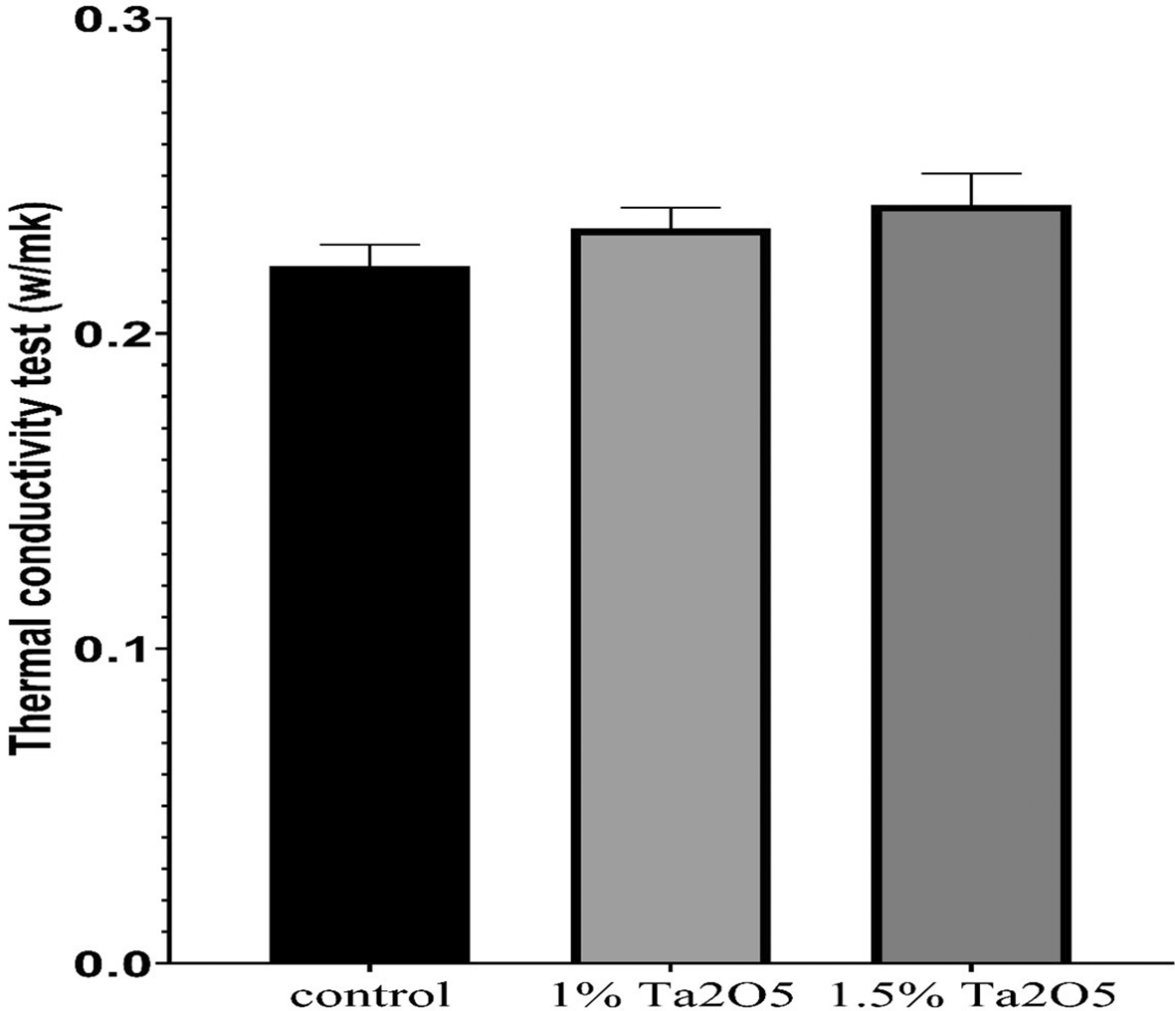
Bar chart of the mean and standard deviation of thermal conductivity for studied groups.

Tear strength test results

The Shapiro-Wilk test revealed a normal distribution of data around the mean (P value > 0.05) ( Table 1).

**Table 1.** Shapiro-wilk test of normality.

Shapiro-wilk	Control	1 wt% Ta _2_O _5_	1.5 wt% Ta _2_O _5_
W	0.9510	0.9400	0.9514
P value	0.6803	0.5533	0.6850
Sig	ns	ns	ns

The descriptive statistic revealed an increase in the mean value as the concentration of Ta _2_O _5_ increased, as shown in Table 2.

**Table 2.** Descriptive statistics of tear strength test.

Groups	No.	Min.	Max.	Mean	± SD	± SE	Lower 95% confidence interval of mean	Upper 95% confidence interval of mean
Control	10	22.00	24.00	23.17	0.6165	0.1950	22.73	23.61
1 wt% Ta _2_O _5_	10	24.30	26.80	25.23	0.7747	0.2450	24.68	25.78
1.5 wt% Ta _2_O _5_	10	25.50	27.30	26.25	0.5817	0.1839	25.83	26.67

One-way ANOVA test revealed a highly significant difference in the mean values among all groups (P < 0.01) ( Table 3).

**Table 3.** One-way ANOVA analysis of variance among groups.

	Sum of squares	D.F.	Mean squares	F	P value
Between groups	49.23	2	24.62	56.01	P<0.0001Hs
Within groups	11.87	27	0.4395		
Total	61.10	29			

To choose the type of multiple comparisons, Bonferroni’s multiple comparisons test was used to assess the homogeneity of variances, and Bartlett’s test was used ( Table 4).

**Table 4.** Bartlett’s test for tear strength test.

Bartlett’s statistic	P value	P value summary
0.8184	0.6642	ns

Bonferroni’s multiple comparison tests revealed a highly significant difference between groups (P < 0.01) ( Table 5).

**Table 5.** Bonferroni’s multiple comparisons test for tear strength test.

	M.D.	P value
control vs. 1% Ta _2_O _5_	-2.060	<0.0001 H. sig
control vs. 1.5% Ta _2_O _5_	-3.080	<0.0001 H. sig
1% Ta _2_O _5_ vs. 1.5% Ta _2_O _5_	-1.020	0.0057 ns

### Thermal conductivity test results

The Shapiro-Wilk test revealed a normal distribution of data around the mean (P value > 0.05) ( Table 6).

**Table 6.** Shapiro-wilk test of normality.

Shapiro-wilk	Control	1 wt% Ta _2_O _5_	1.5 wt% Ta _2_O _5_
W	0.9318	0.8944	0.8880
P value	0.4656	0.1901	0.1612
Sig	ns	ns	Ns

The descriptive statistic revealed an increase in the mean value as the concentration of Ta _2_O _5_ increased, as shown in Table 7.

**Table 7.** Descriptive statistics of the thermal conductivity test.

Groups	No.	Min.	Max.	Mean	± SD	± SE	A lower 95% confidence interval of the mean	Upper 95% confidence interval of mean
Control	10	0.2110	0.2300	0.2214	0.006851	0.002166	0.2165	0.2263
1 wt% Ta _2_O _5_	10	0.2180	0.2410	0.2333	0.006567	0.002077	0.2286	0.2380
1.5 wt% Ta _2_O _5_	10	0.2220	0.2530	0.2408	0.009887	0.003126	0.2337	0.2479

One-way ANOVA test revealed a highly significant difference in the mean values among all groups (P < 0.01) ( Table 8).

**Table 8.** One-way ANOVA analysis of variance among groups.

	Sum of squares	D.F.	Mean squares	F	P value
Between groups	0.001918	2	0.0009588	15.32	P<0.0001Hs
Within groups	0.001690	27	6.260e-005		
Total	0.003608	29			

Bonferroni’s multiple comparisons test was used to assess the homogeneity of variances, and Bartlett’s test was used to choose the type of multiple comparisons ( Table 9).

**Table 9.** Bartlett’s test for thermal conductivity test.

Bartlett’s statistic	P value	P value summary
1.846	0.3974	Ns

Bonferroni’s multiple comparisons tests revealed a highly significant difference between the control and experimental groups (P < 0.01), except there was No significant difference between 1 wt% group and 1.5 wt% group at (P > 0.05) ( Table 10).

**Table 10.** Bonferroni’s multiple comparisons test for thermal conductivity test.

	M.D.	P value
control vs. 1% Ta _2_O _5_	-0.01190	0.0070 Hs
control vs. 1.5% Ta _2_O _5_	-0.01942	<0.0001 Hs
1% Ta _2_O _5_ vs. 1.5% Ta _2_O _5_	-0.007520	0.1286 ns

## Discussion

### Fourier transform infrared spectroscopy

Both before and after the addition of Ta _2_O _5_ nanoparticles, FTIR analyses were carried out. There was no chemical reaction since the spectral range did not change before or after the addition. Fillers and silicone interact to produce the main interaction in this instance, which is characterized as a physical reaction (hydrogen bond or Van der Waals bond). This interaction showed up as a modest alteration in the light transmittance of the silicone matrix and a change in the vibration of preexisting bonds. This demonstrates that since no novel chemical substance was created. ^22^

### Tear strength test

As compared to the control group, the results of the tear strength test showed improvements in tear strength for both experimental groups (1 wt% and 1.5 wt% Ta _2_O _5_), and its proportion raised in direct proportion to the percentage increase (highly significant). Thus, the alternate theory was approved. By forming three-dimensional networks inside the polymer matrix, nanoparticles can physically trap certain polymer chains, resulting in filler meshes inside the polymer matrix. The contact between the nanoparticles and the polymer matrix prevents the polymer chains from moving against the nanoparticles or against each other. Consequently, the density and tear strength increase. ^19^

Rubber materials possess the capability to dissipate strain energy at the point of crack development. Consequently, when the break propagates, nanoparticles may distribute their energy, so enhancing the material’s resistance to rupture. ^22^

### Thermal conductivity test

The thermal conductivity test findings indicated improvements in thermal conductivity for both experimental groups (1 wt% and 1.5 wt% Ta _2_O _5_) relative to the control group, with the proportionate increase corresponding directly to the percentage increase. The null hypothesis was dismissed.

The substantial rise in thermal conductivity values may result from the progressive interaction of particles forming a network arrangement termed heat conductive pathways. This channel facilitates heat flow from one side of the specimen to the other, overcoming the insulating properties of the polymer. Consequently, the polymer will exhibit elevated heat conductivity. ^23^

Furthermore, this phenomenon could be explained by the compact structure of composites. A compact structure of composites with superior heat conductivity over pure polymers is produced as the particles are added to the matrix, taking on the role of gaps and reducing the free volume (air-filled voids). ^23^

## Conclusions

The addition of Ta _2_O _5_ powder to VST-50F maxillofacial silicon elastomer will improve the tear strength and heat conductivity of silicon, and this improvement appears to be concentration-dependent, according to the study’s limits. Ta _2_O _5_ nanoparticles could be added to colored VST-50F RTV silicone elastomers to investigate their effects and assess how they affect the bacterial biofilm’s adherence to the silicones in the maxillofacial region. Another idea that might be investigated is assessing the artificial aging of VST-50F RTV maxillofacial silicone following the inclusion of Ta _2_O _5_ Nano powder.

## Ethics and consent

Ethical approval and consent were not required.

## Data Availability

figshare: “ *The influence of tantalum oxide nanoparticle on thermal conductivity and tear strength after addition on maxillofacial silicone”.* Doi: https://doi.org/10.6084/m9.figshare.28060640.v1. ^24^ This project contains the following underlying data:
• FTIR • Particle size analyzer of Ta _2_O _5_
• raw data of thermal conductivity and tear strength • pictures Data are available under the terms of the Creative Commons Zero “No rights reserved” data waiver (CC0 1.0 Public domain dedication).
