# Supplementary material for: The influence of tantalum oxide nanoparticle on thermal conductivity and tear strength after addition on maxillofacial silicone
Source: F1000Res. 2025 Apr 22;14:189. Originally published 2025 Feb 11. [Version 3] doi: 10.12688/f1000research.159001.3 (PMC12498509; doi:10.12688/f1000research.159001.3)
Supplement: Peer review history

# Reviewer report: Reviewer response for version 3

**DOI:** 10.5256/f1000research.180408.r379778

**Published:** 2025-05-02

**Authors:** Radhwan H. Hasan

**Affiliations:** 1 Mosul University, Mosul, Iraq

The researchers made all the suggestions that aim to raise the level of research.

Is the work clearly and accurately presented and does it cite the current literature?

Yes

If applicable, is the statistical analysis and its interpretation appropriate?

Partly

Are all the source data underlying the results available to ensure full reproducibility?

Yes

Is the study design appropriate and is the work technically sound?

Partly

Are the conclusions drawn adequately supported by the results?

Yes

Are sufficient details of methods and analysis provided to allow replication by others?

Yes

Reviewer Expertise:

prosthodontics , dental material

I confirm that I have read this submission and believe that I have an appropriate level of expertise to confirm that it is of an acceptable scientific standard.

# Reviewer report: Reviewer response for version 2

**DOI:** 10.5256/f1000research.179064.r371388

**Published:** 2025-03-21

**Authors:** Radhwan H. Hasan

**Affiliations:** 1 Mosul University, Mosul, Iraq

The researcher did not complete all the points mentioned in the previous report.

Is the work clearly and accurately presented and does it cite the current literature?

Yes

If applicable, is the statistical analysis and its interpretation appropriate?

Partly

Are all the source data underlying the results available to ensure full reproducibility?

Yes

Is the study design appropriate and is the work technically sound?

Partly

Are the conclusions drawn adequately supported by the results?

Yes

Are sufficient details of methods and analysis provided to allow replication by others?

Yes

Reviewer Expertise:

prosthodontics , dental material

I confirm that I have read this submission and believe that I have an appropriate level of expertise to confirm that it is of an acceptable scientific standard, however I have significant reservations, as outlined above.

# Response

**Published:** 2025-03-25

**Authors:** saif hablalmateen

**Affiliations:** prosthodontic, University of Baghdad, Baghdad, Baghdad Governorate, Iraq

Thanks for you Dr.

 can you mentioned which points didn't complete

# Response

**Published:** 2025-04-07

**Authors:** RADHWAN HIMMADI

**Affiliations:** Prosthodontics, mosul university, Mosul, Iraq

You can read the report carefully and apply its paragraphs.

# Reviewer report: Reviewer response for version 1

**DOI:** 10.5256/f1000research.174668.r366042

**Published:** 2025-02-24

**Authors:** Dr.Manar E. Al-Samaray

**Affiliations:** 1 Mustansiriyah University, Baghdad, Iraq

Dear Editor

 This study is conducted to estimate the influence of addition tantalum oxide nanoparticle on thermal conductivity and tear strength after addition on maxillofacial silicone. The manuscript entitled " *The influence of tantalum oxide nanoparticle on thermal conductivity and tear strength after addition on maxillofacial silicone*" contains indexable content and may be accepted after revision by the respected authors:

**Abstract**

1. Kindly mention the type of silicone, i.e.: RTV in the abstract.
2. Please report the numbers in Ta2O5 as subscripted numbers (Ta _2_O _5_).

**Introduction**

1. The paragraphs should be written coherently.
2. Previous research is missing.
3. Kindly report the abbreviation meaning when its mentioned the first time, then, it can be written as an abbreviation.
4. Please mention the advantages/disadvantages as paragraphs not as separated not organized sections.
5. Kindly discuss the effect of apatite layer formation on tear strength.

**Materials and Methods**

1. Kindly report the model, design, and manufacturer for each device or machine used in the study.
2. Regarding the tear strength test, is the force unit N/mm or KN/mm?
3. Abbreviations can not be reported at the beginning of the sentence.

**Results**

 Kindly report the change in peak intensity in the FTIR results.

**Discussion**

1. Kindly begin the discussion section with the aim of your study.
2. Try to rewrite the discussion as one section with multiple paragraphs

Is the work clearly and accurately presented and does it cite the current literature?

Yes

If applicable, is the statistical analysis and its interpretation appropriate?

Yes

Are all the source data underlying the results available to ensure full reproducibility?

Yes

Is the study design appropriate and is the work technically sound?

Yes

Are the conclusions drawn adequately supported by the results?

Yes

Are sufficient details of methods and analysis provided to allow replication by others?

Yes

Reviewer Expertise:

Prosthodontist

I confirm that I have read this submission and believe that I have an appropriate level of expertise to confirm that it is of an acceptable scientific standard.

# Response

**Published:** 2025-02-24

**Authors:** saif hablalmateen

**Affiliations:** prosthodontic, University of Baghdad, Baghdad, Baghdad Governorate, Iraq

Dear Dr. Manar E. Al-Samaray,

 Thanks for your comments 

 1- About the Ta _2_o _5 _we write it as this formolation

 2- The unite of tear is kn/m 

 3- About the abbreviation we write all it with complete meaning 

 4- About the design the device i send all the figure and detail in figshare document

# Response

**Published:** 2025-03-03

**Authors:** saif hablalmateen

**Affiliations:** prosthodontic, University of Baghdad, Baghdad, Baghdad Governorate, Iraq

Dear Dr. 

 1- I mention the type of silicone 

 2- I type tantalum oxide in this formula (ta _2_O _5_)

 3- The paragraphs written coherently

 4- All abbreviation firstly write the meaning

 5- Mentioned all advantages or disadvantages as paragraph

 6- All design, and details about machine or device I mentioned in during the paragraphs

 7- N/mm the unite of force in the tear strength

 8- Not begin with abbreviations

 9- There is no chemical change in the FTIR

 10- Start the discussion with the aim of study and write it as on section

# Reviewer report: Reviewer response for version 1

**DOI:** 10.5256/f1000research.174668.r366037

**Published:** 2025-02-14

**Authors:** Radhwan H. Hasan

**Affiliations:** 1 Mosul University, Mosul, Iraq

1. **Strengths of the Study**:

    - The study exhibits strong clarity in its methodology, which is crucial for reproducibility and credibility. Detailed explanations of material selection, experimental setups, and testing procedures make the study transparent.

    - Incorporating statistical tests such as one-way ANOVA and Bonferroni's multiple comparisons enhance the validity of the findings. By using rigorous statistical methods, the study ensures that the results are not due to random chance, which adds robustness to the conclusions.

 2. **Weaknesses of the Study**:

    - **Lack of Discussion**: The paper falls short in offering an in-depth discussion of the results. This omission prevents readers from understanding how the findings fit into the broader context of existing research. For instance, if previous studies on similar nanoparticle composites show consistent or conflicting results, this comparison should be highlighted.

    - **Sample Size Justification**: Although the paper includes 60 specimens, it neglects to justify this sample size or present a power analysis. Sample size justification is crucial for determining whether the study could adequately detect significant effects. Without this, the results could be questioned for not having enough statistical power.

    - **Insufficient Interpretation**: The conclusion that tantalum oxide nanoparticles enhance tear strength and thermal conductivity is made without enough depth. The practical implications for maxillofacial prosthetics or the mechanisms underlying the enhanced properties are not thoroughly explored. Discussing potential applications or limitations would strengthen the argument.

 3. **Validity of Arguments**:

    - **Hypothesis Testing**: The study sets out with clear null and alternative hypotheses, important for guiding research. Nonetheless, the discussion fails to re-evaluate these hypotheses in light of the results, weakening the paper’s narrative arc. Explicitly stating how the findings support or refute the hypotheses would have added clarity and strength.

    - **Experimental Design**: The study’s design appears robust with appropriate control and experimental groups. However, the paper would benefit from an explanation of why 1wt% and 1.5wt% were chosen as the specific weight percentages for the nanoparticles to ensure the rationale behind these concentrations is sound and relevant.

 4. **Recommendations**:

    - **Expand Discussion Section**: Enhancing the discussion to interpret findings deeply and link them to previous research will offer a richer context. For example, comparing this study’s results with others that used different nanoparticles or weight percentages could provide insights into the specific role of tantalum oxide.

    - **Justification of Sample Size**: Including a power analysis or rationale behind choosing 60 specimens will reinforce the study’s credibility. Providing outcomes from a pilot study could also substantiate the chosen sample size.

    - **Addressing Practical Implications**: Discussing how the improved tear strength and thermal conductivity affect the usability and performance of maxillofacial silicone prosthetics can make the paper more impactful. It could explore potential benefits for patients or manufacturing processes, making the results more relevant for practical applications.

 **Concrete Examples**:

 - **Lack of Discussion**: A similar study on alumina nanoparticles showed increased tear strength in maxillofacial silicones (reference: Elmorsy et al., 2019). Comparing findings could provide insights into whether tantalum oxide offers unique advantages or if any observed differences are due to specific properties of the nanoparticles used.

 - **Sample Size Justification**: For example, a power analysis in a related study on biomaterials may have shown that a minimum sample size of 50 is required to detect a 10% difference in material properties with 80% power. Stating such specifics strengthens the study's design.

 By addressing these points, the paper can enhance the validity of its arguments and contribute more effectively to the field of maxillofacial prosthetics.

Is the work clearly and accurately presented and does it cite the current literature?

Yes

If applicable, is the statistical analysis and its interpretation appropriate?

Partly

Are all the source data underlying the results available to ensure full reproducibility?

Yes

Is the study design appropriate and is the work technically sound?

Partly

Are the conclusions drawn adequately supported by the results?

Yes

Are sufficient details of methods and analysis provided to allow replication by others?

Yes

Reviewer Expertise:

prosthodontics , dental material

I confirm that I have read this submission and believe that I have an appropriate level of expertise to confirm that it is of an acceptable scientific standard, however I have significant reservations, as outlined above.

# Response

**Published:** 2025-02-14

**Authors:** saif hablalmateen

**Affiliations:** prosthodontic, University of Baghdad, Baghdad, Baghdad Governorate, Iraq

I wish to let the reviewer know that we also want to add only reference number 24 at the end of the Fabrication and conditioning of the specimens section of the article besides the Reference number 18,19 that indicate the number of sited reference.

 We are in talks with the copy editing team to update the same at the earliest

# Response

**Published:** 2025-02-24

**Authors:** saif hablalmateen

**Affiliations:** prosthodontic, University of Baghdad, Baghdad, Baghdad Governorate, Iraq

thanks dear dr. for your comments 

 1- about the sample size we use 10 sample for control group and 10 sample for 1% and 10 samples for 1.5% for each test and from pilot study we choose the best percent of tantalum oxide nanoparticle

 2- about the discussion we mention briefly about the improvement on the tear and thermal test
